# MetaFlux: Meta-learning global carbon fluxes from sparse spatiotemporal observations

**DOI:** 10.1038/s41597-023-02349-y

**Published:** 2023-07-11

**Authors:** Juan Nathaniel, Jiangong Liu, Pierre Gentine

**Affiliations:** 1 Department of Earth and Environmental Engineering, Columbia University, New York, NY 10027 USA; 2 Climate School, Columbia University, New York, NY 10027 USA

**Keywords:** Carbon cycle, Hydrology

## Abstract

We provide a global, long-term carbon flux dataset of gross primary production and ecosystem respiration generated using meta-learning, called *MetaFlux*. The idea behind meta-learning stems from the need to learn efficiently given sparse data by learning how to learn broad features across tasks to better infer other poorly sampled ones. Using meta-trained ensemble of deep models, we generate global carbon products on daily and monthly timescales at a 0.25-degree spatial resolution from 2001 to 2021, through a combination of reanalysis and remote-sensing products. Site-level validation finds that MetaFlux ensembles have lower validation error by 5–7% compared to their non-meta-trained counterparts. In addition, they are more robust to extreme observations, with 4–24% lower errors. We also checked for seasonality, interannual variability, and correlation to solar-induced fluorescence of the upscaled product and found that MetaFlux outperformed other machine-learning based carbon product, especially in the tropics and semi-arids by 10–40%. Overall, MetaFlux can be used to study a wide range of biogeochemical processes.

## Background & Summary

Data sparsity is a prevalent challenge in climate science and ecology. For example, *in-situ* observations tend to be spatially and temporally sparse due to sensor malfunctions, limited sensor locations, or non-ideal climate conditions such as persistent cloud cover. Consequently, understanding many climate processes can be difficult because the data do not capture the full natural variability in both space and time. FLUXNET2015 is a global network of eddy-covariance stations that captures carbon, water, and energy exchanges between the atmosphere and biosphere and provides high-quality ecosystem-scale observations spanning many climate and ecosystem types^1^. However, its coverage is neither continuous nor temporally dense, especially in the years prior to 2000^1^. Furthermore, its distribution across climate zones is not balanced, with only around 8% and 11% of the current operational stations located in the tropics and semi-arid regions, which are regions of critical importance for the global carbon cycle^2^. For instance, there is increasing evidence that most of the global interannual carbon variability can be attributed to the semi-arid ecosystems in the southern hemisphere^3^. Thus, the lack of high-resolution observations in these data-sparse, yet important areas may inhibit our overall understanding of the global carbon cycle, especially in light of climate change.

The machine-learning community has tried to tackle the data sparsity problem in many ways, including the development of several few-shot learning approaches^4,5^. One of these is the meta-learning approach that “learns how to learn from different tasks”. The idea behind this learning paradigm closely resembles how humans learn: we extract high-level features from previously learned tasks to quickly solve new problems. For instance, we can memorize a new person’s face with very few samples because we understand how a face should look after seeing many other faces. Although applications of meta-learning have been limited^6,7^, there has been a growing popularity in the applied sciences^8,9^. However, as far as we know, there is little work being done on the use of meta-learning in climate and environmental sciences, especially with regards to sparse and extreme spatiotemporal observations. In addition, to the best of our knowledge, there has been no upscaling effort to date that uses an ensemble of meta-trained deep models to produce a spatiotemporally continuous climate product from sparse observations. And given the importance of carbon fluxes in diagnosing the earth’s changing climate^10,11^, there is a growing need to have a globally continuous, high-resolution dataset that best represents critical regions that, unfortunately, tend to have few data points.

To bridge these gaps, we aim to (i) evaluate the performance of meta-learning in environments where spatiotemporal information is sparse, (ii) check its robustness when predicting extreme cases that are critical to the carbon cycle, and (iii) upscale point data to a globally continuous map using an ensemble of meta-trained deep models. In particular, we focus on gross primary production (GPP) and ecosystem respiration (*R*_*eco*_) as specific applications of the broader terrestrial carbon cycle. The upscaled product is resolved at 0.25-degree spatial resolution, spanning either at daily or monthly temporal resolutions between the years 2001 and 2021. Preliminary analysis shows that our global product (“MetaFlux”) is internally consistent in terms of its seasonality, interannual trend, and variability, and has high correlation with satellite-based solar-induced fluorescence (SIF) – a proxy for photosynthesis – when compared with other data-driven products, especially in the tropics and semi-arid critical regions.

**Fig. 1:**
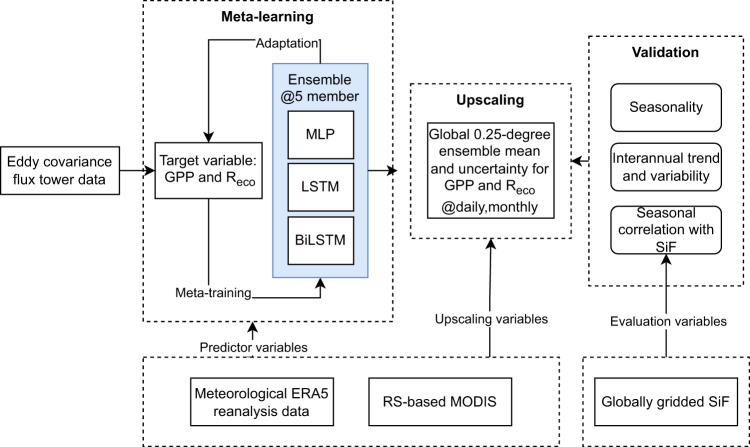
Schematic diagram of our MetaFlux methodology from the meta-learning phase that meta-trains GPP and *R*_*eco*_ from FLUXNET2015 eddy covariance flux tower data using station-level ERA5 reanalysis and RS-based MODIS predictors. The meta-trained and validated ensemble is then used to upscale a global 0.25-degree GPP and *R*_*eco*_ at daily and monthly timescale between the years 2001 and 2021. Finally, the ensemble global mean and uncertainty estimates are validated and compared with evidences from the literature.

Finally, the dataset is freely accessible in Zenodo at 10.5281/zenodo.7761881^12^, while the meta-learning code can be reproduced and extended from https://github.com/juannat7/metaflux with example notebooks to apply our approach to your specific use cases. The overall methodology is summarized in Fig. 1.

## Methods

### Meta-learning: learning how to learn

**Fig. 2:**
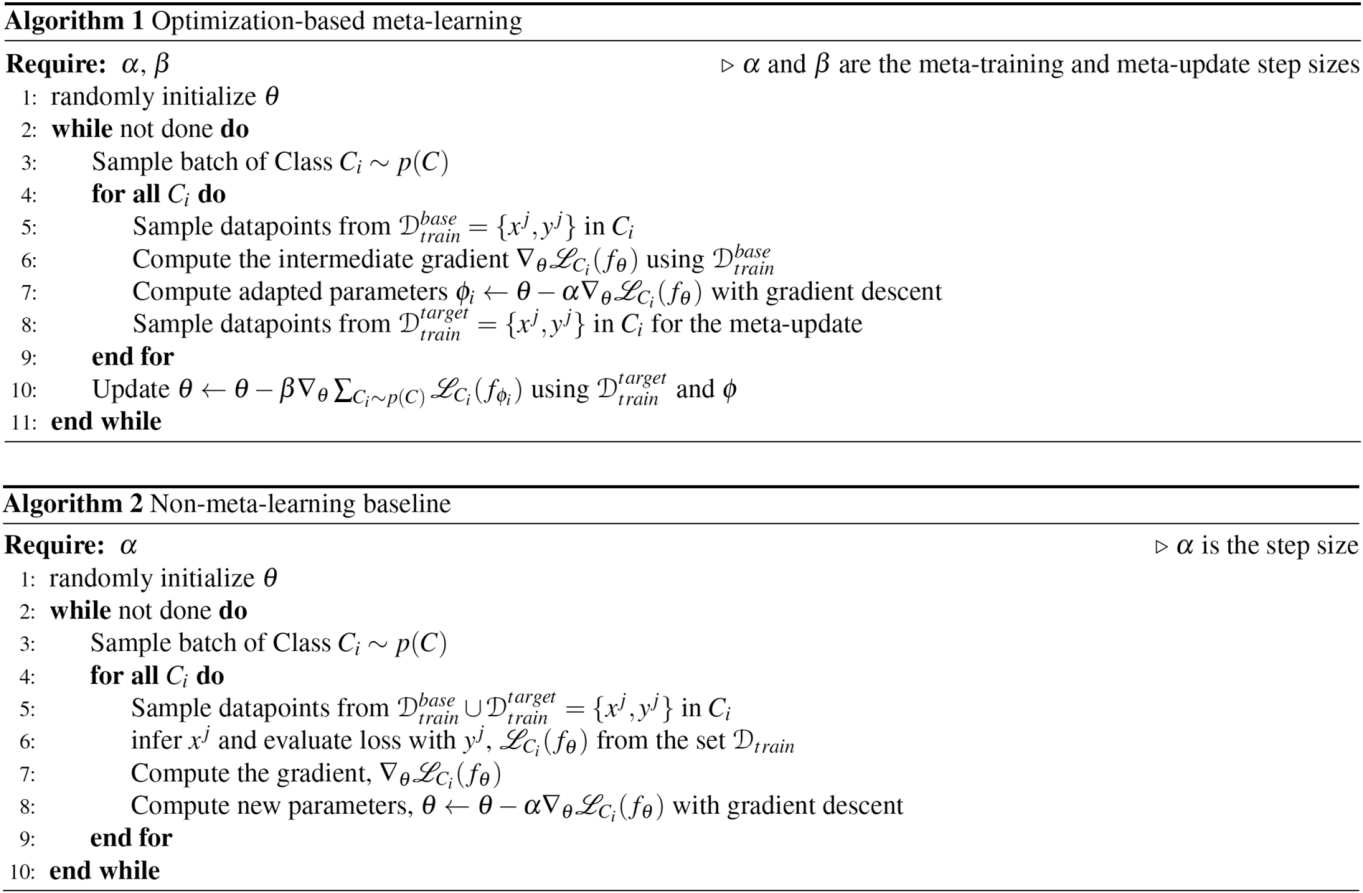
Algorithm for both optimization-based meta-learning and its non-meta-learning counterpart.

**Fig. 3:**
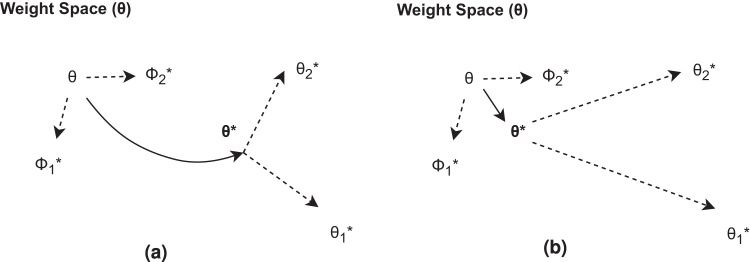
Schematic diagram of our (**a**) meta-learning approach that is meta-trained on data-abundant tasks to obtain a set of *ϕ*, and fine-tuned on data-sparse tasks to get θ*; (**b**) baseline algorithm that is not meta-trained. The optimal θ* in the latter case tends to be biased towards data-abundant tasks as represented by the gradient sets ${\phi }_{n}^{* }$.

**Fig. 4:**
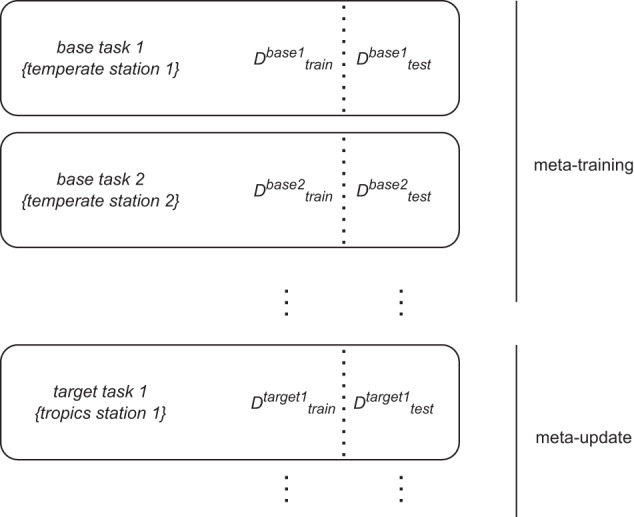
Meta-learning datasets where base tasks are used for meta-training and target tasks for meta-update. The subscripts *train* and *test* are used to train and evaluate the model in each of the two meta-learning steps. Target tasks are selected by randomly sampling half of the FLUXNET2015 stations in the tropical and semi-arid regions, which are sparse, and one station from each plant functional type (PFT) including those in the cropland and boreal regions. By extension, the base tasks consist of the remaining stations.

Meta-learning is a machine-learning paradigm that trains a model to learn new tasks from sparse data efficiently^13^, leveraging information that comes from tasks with more available data. In general and as illustrated in Fig. 3a, meta-learning involves two stages: a meta-training and a meta-update stage. During meta-training, the model, *f*_*θ*_ (a function *f* that is parameterized by *θ*), proposes intermediate parameters, *ϕ*, that minimize the loss of base tasks^14^. In the meta-update step, the model fine-tunes its parameter, *θ*, given *ϕ* and the target tasks^14^, seeking the optimal parameter θ*. We define target tasks as a collection of stations in data-sparse regions, including the tropics, semi-arid regions, and representative stations in each ecoregion defined by plant functional types (PFTs), while the base tasks consist of the complement of the former (i.e. stations in data-abundant regions). Given the two-step gradient update procedures in meta-training and meta-update loops, the optimal parameters θ*, are not biased toward data-abundant base tasks, as illustrated in Fig. 3a, whereas in the baseline case (Fig. 3b), the optimal learned parameters would be biased toward data-abundant tasks as each data point contributes similarly to model’s learning. The details on how the data is split, including how the base (*D*^*base*^) and target (*D*^*target*^) datasets are divided for training and testing, are provided in the “Training setup” section below and illustrated in Fig. 4. For this work, we use an optimization-based meta-learning approach that is adapted from the model-agnostic meta-learning (MAML)^13^ as detailed in Fig. 2a.

### Differentiable learners

Next, we will discuss the different deep learning models used for this work, including a multilayer perceptron (MLP), long-short term memory (LSTM), and bi-directional LSTM (BiLSTM).

#### Multilayer perceptron (MLP)

MLP or the feedforward artificial neural network is a fully connected deep model that can capture nonlinear relationships between inputs and the response variable^15^. Generally, an MLP consists of the input and output layers with several hidden layers and is activated by a set of nonlinear functions. In this paper, we use the Leaky Rectified Linear Unit (LeakyReLU) which is formulated in Eq. 1, where *α* controls the extent of “leakiness” in the negative x direction. The MLP model receives instantaneous weather data and vegetation index.

1 $$R(x)=\left\{\begin{array}{cc}x & x > 0\\ \alpha x & \,x < =0\end{array}\right.$$

#### Long-Short Term Memory (LSTM)

Time-series representing environmental processes tends to be strongly autocorrelated in time^16^ and Recurrent Neural Networks (RNN) were first introduced to solve this issue. The LSTM model was then proposed by^17^ to address the issues of vanishing and exploding gradients commonly observed in RNNs. The model can preserve long-term dependencies of sequential data through its gated structure that controls how information flows across cells. It is able to leverage association across multiple timesteps to inform inferential tasks where time dependency is present and significant. This can be especially useful to represent water stress, for instance, that depends not only on current daily precipitation but also on previous time steps of precipitation (water supply) and evaporative demand (temperature, radiation, humidity).

#### Bi-directional LSTM (BiLSTM)

The BiLSTM model is trained on both forward and backward timesteps to best estimate the value at the current timestep, *t*^18^, similar to reanalysis products (compared to weather forecasts that only use past information like LSTMs). BiLSTM has been used in cases where the past is as important as the future contexts^19^. The equations describing a BiLSTM cell are similar to that in LSTM with a slight modification in the hidden state representations that have to capture the forward and backward timesteps.

### Training setup

We train an ensemble of meta-trained deep networks. The purpose of training an ensemble is to quantify uncertainty and reduce the bias of each individual model^20^. The final model architecture and hyperparameters, including batch size and learning rates, are determined after performing a k-fold cross validation (k = 5) on the training set. In all, we use a 3-layer MLP with a hidden size of 350. We replace the first layer with either the LSTM or BiLSTM modules for the LSTM and BiLSTM models respectively to capture temporal features prior to the final prediction layer. The optimized ensemble is trained by minimizing the mean squared error (MSE). We train our models to estimate GPP and *R*_*eco*_ at daily and monthly timescale across 206 FLUXNET2015 sites^1^ (retrievable from https://fluxnet.org/data/fluxnet2015-dataset/) using a combination of meteorological and remote sensing inputs including precipitation, air temperature at 2-meter (Ta), vapor pressure deficit (VPD), and incoming short-wave radiation from ERA5 reanalysis data^21^ (retrievable from https://cds.climate.copernicus.eu/) and leaf area index (LAI) from the Moderate Resolution Imaging Spectroradiometer (MODIS) product^22^ (retrievable from https://modis-land.gsfc.nasa.gov/). We retrieve the associated time-series closest to each tower site. In particular, we use the night-time partitioning methods for GPP and *R*_*eco*_ and match each flux record with reanalysis and remote sensing data corresponding to the station of interest. Aside from the target variable, we perform a z-normalization of the inputs to improve the learning process (Eq. 2).

2 $$z-normalized\;X=\frac{X-E(X)}{{\sigma }_{X}}$$

where *E*(*X*) is the estimated expected value of the variable X on our training data, and *σ*_X_ the corresponding sample standard deviation.

Next, we define class *C*_*i*_ as a set of batches consisting of input variables and target flux observations, with *i* denoting the index of batches of size 256. The definition of batch differs between linear MLP and time-series models, such as LSTM and BiLSTM. A batch, in the linear MLP case, refers to a collection of instantaneous data points, while in time-series models, it corresponds to a set of 30-day continuous data points. The choice of a 30-day window to form a single data point in the latter case is to better capture seasonal water stress^23^. We construct *D*^*target*^ by randomly selecting half of the stations in the tropical and semi-arid regions (defined by the Köppen classification^24^, retrievable from http://www.gloh2o.org/koppen/), which are sparse, and one station from each plant functional type (PFT) including those in the cropland and boreal areas^25^. By extension, the *D*^*base*^ consist of stations that are complement to *D*^*target*^. Each dataset is divided into training (*D*_*train*_) and testing (*D*_*test*_) sets with an 80:20 split ratio. As the term suggest, the former is used to train the model *f*_*θ*_, while the latter is used to validate the performance of the model. Overall, the set of all possible datasets, *D*, includes $\left({D}_{train}^{base},{D}_{test}^{base},{D}_{train}^{target},{D}_{test}^{target}\right)$. Figure 4 illustrates how the dataset for meta-learning is constructed, with base tasks data being used for meta-training and that from target tasks for meta-update steps. We meta-train three models: MLP, LSTM, and BiLSTM, with an ensemble of five members each; where their individual weights are randomly initialized.

The non-meta-learning baseline uses identical architectures and hyperparameters, but the models do not have a meta-update outer loop (see Fig. 2b). This learning paradigm is similar to a single-step gradient descent learning approach^26^ where we backpropagate the gradient of the loss function to update *f*_*θ*_. This learning mode, however, can be biased to representations of tasks that have a lot more data. To ensure that a similar data structure is used in the baseline case, we compile $\left({D}_{train}^{base},{D}_{train}^{target}\right)$ as the training set and $\left({D}_{test}^{base},{D}_{test}^{target}\right)$ as the testing set.

### Upscaling of global products

For the upscaling portion of this work, we use a similar set of meteorological and remote sensing inputs as during training at either the daily or monthly timesteps. Since VPD is not available in the existing ERA5 catalogue, we estimate it from air and dewpoint temperatures through the saturated (SVP) and actual vapor pressure (AVP) relation: VPD = SVP-AVP, which are both functions of Ta and dewpoint temperatures (Td). Finally, the spatial resolution of the resulting data inputs is harmonized to 0.25-degree using an arithmetic averaging. The final product has four variables, including the ensemble mean estimate of GPP and *R*_*eco*_, and its uncertainty as captured by the standard deviation.

### Evaluation on the site and global level

First, we compare the performance of meta-trained versus non-meta-trained models in terms of their RMSE scores on the testing sets. In addition, we evaluate how robust meta-trained models are in predicting extreme fluxes. This is done by selecting GPP or *R*_*eco*_ fluxes that exceed a predefined z-normalized threshold, *t*, that we vary between 1.0 and 2.0 (i.e. higher threshold means more extreme observations away from the mean value).

Next, we evaluate the upscaled product by analyzing its seasonality and interannual trends across climate zones. Thereafter, we compute the interannual variability using the interannual coefficient of variation (CV; Eq. 3) at the pixel level:

3 $$CV=\frac{\sigma }{\mu }$$

where *σ* and *μ* are the interannual standard deviation and mean, respectively.

Finally, the Pearson correlation coefficient between GPP and solar-induced fluorescence (SIF) from CSIF^27^ (retrievable from https://figshare.com/articles/dataset/CSIF/6387494) and TROPOMI SIF^28^ (retrievable from http://ftp.sron.nl/open-access-data-2/TROPOMI/tropomi/sif/v2.1/l2b/) is calculated across climate zones on a monthly timescale, for the periods 2001–2018 and 2019–2020, respetively. To benchmark our product, we compare our GPP-SIF correlation estimate, r(*GPP*_*metaflux*_, SIF) with that of Fluxcom data-driven product^29^, r(*GPP*_*fluxcom*_, SIF), between the years 2001 and 2020. Generally-speaking, a higher correlation corresponds to a better GPP estimate, though this is not always the case as different ecosystem regimes and physiological characteristics may manifest different associative patterns^30^.

## Data Records

**Fig. 5:**
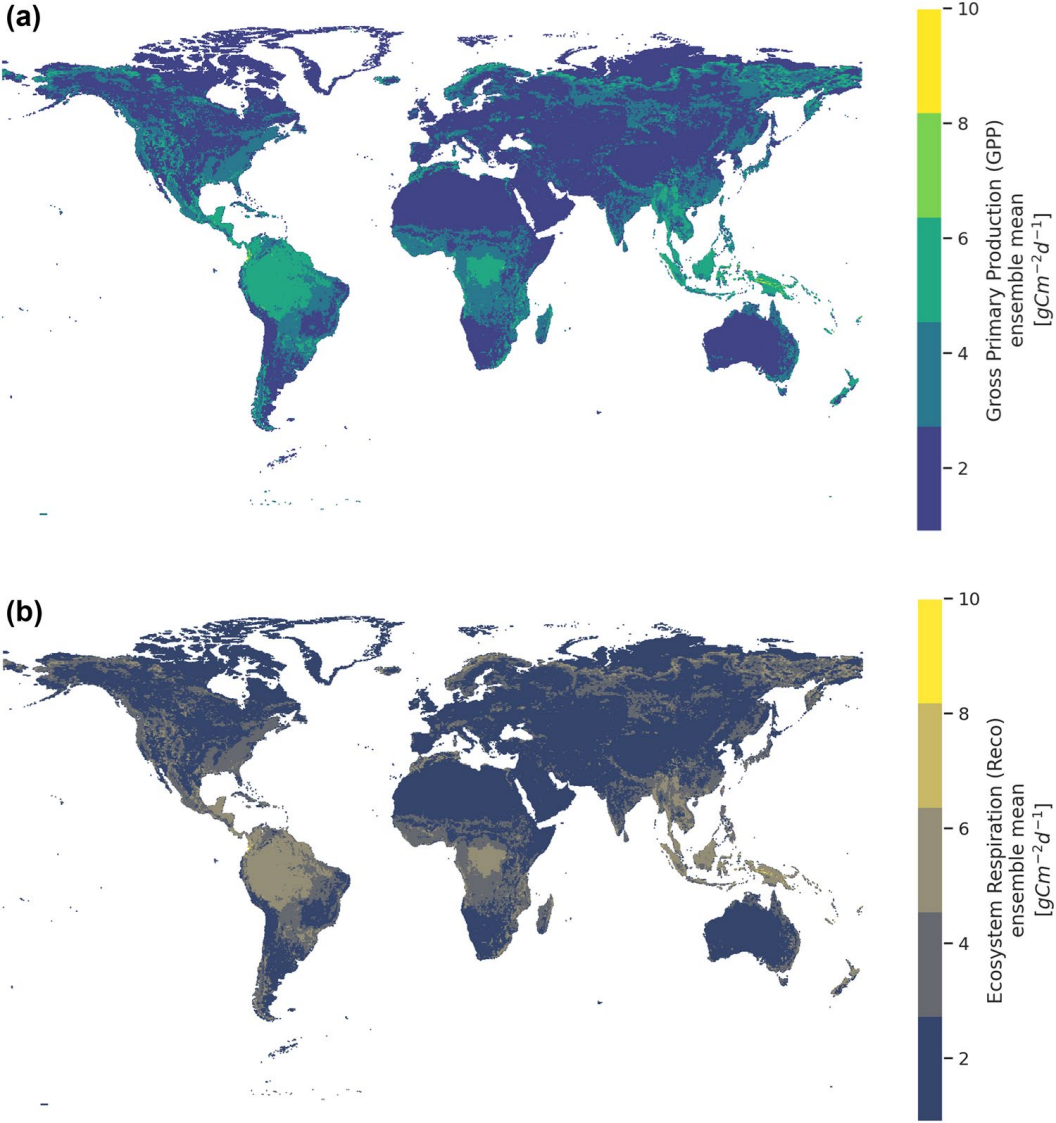
Mean ensemble estimate of (**a**) GPP and (**b**) *R*_*eco*_ for the year 2021. Higher estimates of GPP and *R*_*eco*_ are observed in the tropics while lower ones are in the semi-arid regions.

**Fig. 6:**
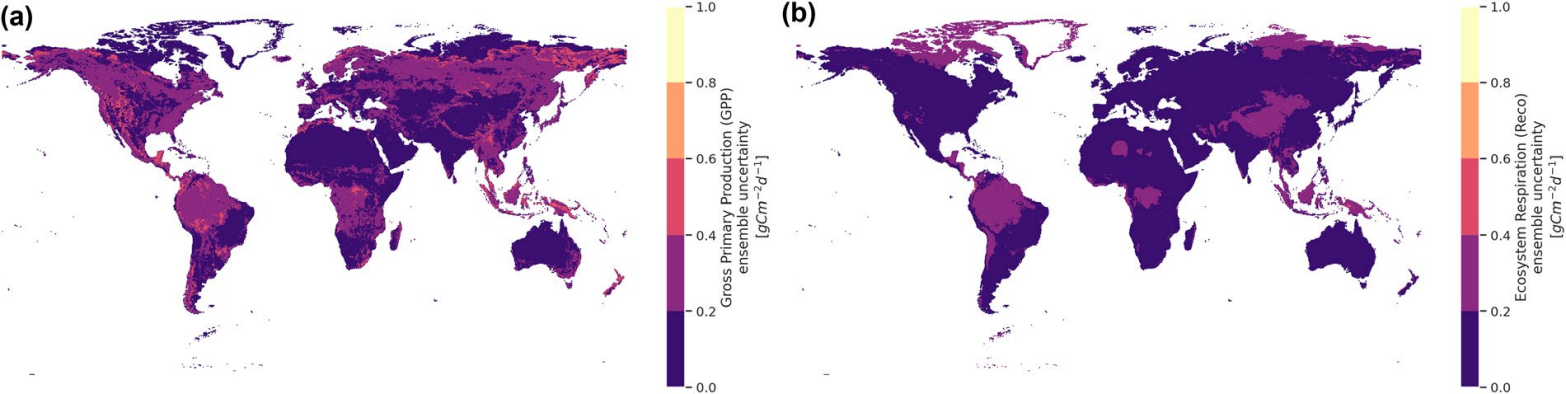
Ensemble uncertainty in terms of the standard deviation of (**a**) GPP and (**b**) *R*_*eco*_ for the year 2021. Generally, GPP has higher uncertainty than *R*_*eco*_, especially in the equator and higher-latitude regions.

The global products amount to around 50GB and are freely accessible in Zenodo at 10.5281/zenodo.7761881^12^. The spatial resolution is 0.25-degree, extending between 90-degree north to 90-degree south, and between 180-degree west and 180-degree east. We mask out cold regions that consist of the Arctic circle and Antarctica. Each Network Common Data Form (NetCDF) file contains four variables: GPP, *R*_*eco*_, GPP_std, *R*_*eco*__std that represent GPP, *R*_*eco*_ ensemble mean and their uncertainties respectively. Temporally, each file is resolved at either the daily or monthly timescale. For instance, Fig. 5 illustrates the annual ensemble mean, while Fig. 6 the ensemble uncertainties of GPP and *R*_*eco*_ for the year 2021. We note that GPP tends to have higher uncertainty than *R*_*eco*_, especially in the equator and higher-latitude regions.

For the daily product, the naming convention for each .nc file is METAFLUX_GPP_RECO_daily_<year><month>.nc; where <year> takes a value between 2001 and 2021 and <month> between 01 and 12 for January and December.

For the monthly product, we perform identical training and upscaling steps but using monthly, rather than daily fluxes, reanalysis, and remote sensing products. The naming convention for each file is METAFLUX_GPP_RECO_monthly_<year>.nc; where <year> takes a value between 2001 and 2021.

## Technical Validation

In this section, we first evaluate our meta-learning approach based on site-level validation RMSE and its robustness to extreme observations. Next, we examine the seasonality, interannual trend and variability, and correlation with independent SIF products.

### Evaluation of meta-learning as a learning framework

#### Convergence and site-level performance

**Fig. 7:**
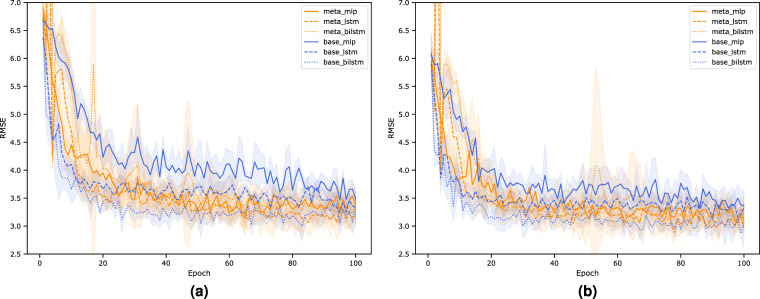
Site-level validation errors for (**a**) GPP and (**b**) *R*_*eco*_ across differentiable models (MLP, LSTM, and BiLSTM) that are meta-trained (orange lines) or not (blue lines). The shaded regions represent the standard deviation of RMSE across 5 model runs and 100 epochs (where a single epoch refers to a complete pass over the entire training dataset). In general, the meta-trained models perform better, and the choice of internal learner matters as demonstrated by the overall lower RMSE of the either LSTM or BiLSTM time models that account for temporal dependency.

As illustrated in Fig. 7 and Table 1, meta-trained deep models generally perform better than their baseline non-meta-trained counterparts. For instance, the validation RMSE of the meta-trained MLP on GPP is 3.13 *gC m*^−2^ *d*^−1^  ± 0.06 as compared to 3.47 *gC m*^−2^ *d*^−1^  ± 0.07 in the baseline case. A similar result is observed for *R*_*eco*_ where the RMSE of the meta-trained MLP is 3.07 *gC m*^−2^ *d*^−1^  ± 0.05 as compared to 3.31 *gC m*^−2^ *d*^−1^  ± 0.07 in the baseline case. In addition, the choice of deep networks matters. Overall, models that incorporate temporal information, i.e. the LSTM and BiLSTM models, perform better than models that do not. In the GPP case, for example, the non-meta-trained BiLSTM model has the lowest validation error of 3.00 *gC m*^−2^ *d*^−1^  ± 0.04, followed by the meta-trained LSTM model with an RMSE of 3.06 *gC m*^−2^ *d*^−1^  ± 0.06. This confirms our physical intuition that water stress, which tends to regulate productivity, builds up over many days to months and thus requires a memory process as captured by the recurrent neural networks. Moreover, plant photosynthesis and respiration can acclimate to the prevailing environmental conditions, such as temperature, light and VPD^31,32^, which tend to be captured more effectively by memory-informed models. Nonetheless, the addition of bi-directionality in the BiLSTM model does not appear to significantly reduce error in the meta-trained models. This can be because the concept of data assimilation from future context has been captured through the process of meta-learning itself or that the signal coming from unidirectional timeseries is sufficiently saturated to parameterize the model. In other words, since our meta-learning approach primarily considers the spatial heterogeneity of the fluxes (e.g., across climate zones and PFTs), this spatial information, along with the temporal signals coming from BiLSTM gradient steps, result in a more unstable learning due to signal oversaturation which is evident from the larger convergence spread across model runs. This can be regularized by considering not just spatial, but also the spatiotemporal heterogeneity in a meta-learning approach^33^, though this will increase the complexity of the algorithm and could potentially limit its extrapolation capacity. This remains the subject of future work.

**Table 1:** Site-level validation RMSE (*gCm*^−2^ *d*^−1^) across differentiable models for GPP and *R*_*eco*_.

Model	GPP		*R*_*eco*_	
	Baseline	Meta-trained	Baseline	Meta-trained
MLP	3.47 ± 0.07	**3.13 ± 0.06**	3.31 ± 0.07	**3.07 ± 0.05**
LSTM	3.25 ± 0.01	**3.06 ± 0.06**	3.13 ± 0.02	**2.89 ± 0.06**
BiLSTM	**3.00 ± 0.04**	3.18 ± 0.07	**2.87 ± 0.04**	3.09 ± 0.12

The numbers in bold correspond to models that have lower RMSE in either the baseline or meta-learning cases.

#### Robustness under extreme conditions

**Fig. 8:**
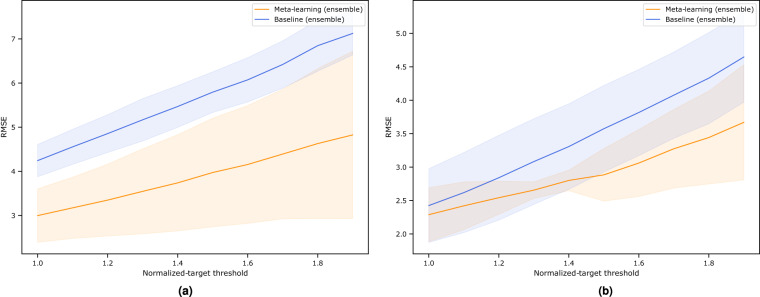
Comparison between the performance of meta-trained and baseline models at an increasing level of extreme (**a**) GPP and (**b**) *R*_*eco*_ daily observations. The y-axis indicates the average RMSE when the ensemble predicts carbon fluxes with z-value greater than the threshold *t*. Overall, we find that the meta-trained ensemble has lower RMSE across increasing extreme threshold than its baseline counterpart.

Making an accurate estimate for extreme cases is especially important in climate science because extreme weather tends to cause catastrophic damages, such as major droughts, wildfires, or plant mortality^34,35^. Fig. 8 illustrates the performance of our meta-trained models under an increasing magnitude of extremes as defined by the z-normalized threshold, *t*. In general, our meta-trained models (orange line) are more robust in predicting extreme cases of observed GPP and *R*_*eco*_ (i.e. lower validation RMSE) than their baseline counterparts (blue line), with a difference of around 1.2 *gC m*^−2^ *d*^−1^ and 0.7 *gC m*^−2^ *d*^−1^ for GPP and *R*_*eco*_ respectively.

If we further examine model performance across climate zones (Tables 2, 3) and select extreme fluxes with a normalized-target threshold, *t*, that is greater than 1.0, we find that our meta-trained models outperform the baselines. The reason why we choose this threshold is to have sufficient extreme observations across climate zones such that a more meaningful comparison can be made. In the GPP case, for example, meta-trained ensemble has lower validation RMSE of 3.78 *gC m*^−2^ *d*^−1^  ± 0.33 (versus 4.10 *gC m*^−2^ *d*^−1^  ± 0.29) and 3.04 *gC m*^−2^ *d*^−1^  ± 0.02 (versus 3.45 *gC m*^−2^ *d*^−1^  ± 0.06) in the semi-arid and tropics, respectively. A similar finding is observed for *R*_*eco*_ where meta-trained ensemble has lower validation RMSE of 2.35 *gC m*^−2^ *d*^−1^  ± 0.04 (versus 2.65 *gC m*^−2^ *d*^−1^  ± 0.06) in the tropics. These results are promising as the representation of both the tropics and semi-arid regions in many upscaled products is often challenging due to the limited number of observations available and the complex, memory-like processes involved. For example, in the semi-arid regions, there is a build-up of time-dependent water stresses^36^, while in the tropics, there is a complex seasonal cycle of leaf flushing and phenology^37,38^. Our approach is superior in its ability to reproduce carbon fluxes in the tropics and semi-arid areas because limited data here are optimally enriched with shared information coming from other data-abundant regions through meta-learning.

**Table 2:** Robustness of meta-trained ensemble when inferring extreme GPP observations across climate zones at the normalized-target threshold, *t* > 1.0 (all units in *gC m*^−2^ *d*^−1^).

Climate zones	Mean extreme GPP	Baseline RMSE	Meta-trained RMSE
Semi-arid	11.48	4.10 ± 0.29	**3.78 ± 0.33**
Continental	11.47	3.62 ± 0.10	**3.61 ± 0.07**
Temperate	11.45	3.89 ± 0.09	**3.71 ± 0.04**
Tropics	10.68	3.45 ± 0.06	**3.04 ± 0.02**

The numbers in bold represent models with lower RMSE between the baseline and meta-learning cases across different climate zones.

**Table 3:** Robustness of meta-trained ensemble when inferring extreme *R*_*eco*_ observations across climate zones at the normalized-target threshold, *t* > 1.0 (all units in *gC m*^−2^ *d*^−1^).

Climate zones	Mean extreme *R*_*eco*_	Baseline RMSE	Meta-trained RMSE
Semi-arid	8.80	1.37 ± 0.10	1.42 ± 0.06
Continental	8.70	2.29 ± 0.06	**2.27 ± 0.05**
Temperate	8.51	2.13 ± 0.08	**2.06 ± 0.06**
Tropics	8.71	2.65 ± 0.06	**2.35 ± 0.04**

The numbers in bold represent models with lower RMSE between the baseline and meta-learning cases across different climate zones.

### Evaluation of meta-learned global data

Now that we have validated our meta-learning framework on the site level, we proceed to evaluate the internal consistency of our upscaled product. This includes the analysis of seasonality, interannual variability, and comparison to SIF as an independent photosynthesis product.

#### Temporal analysis

**Fig. 9:**
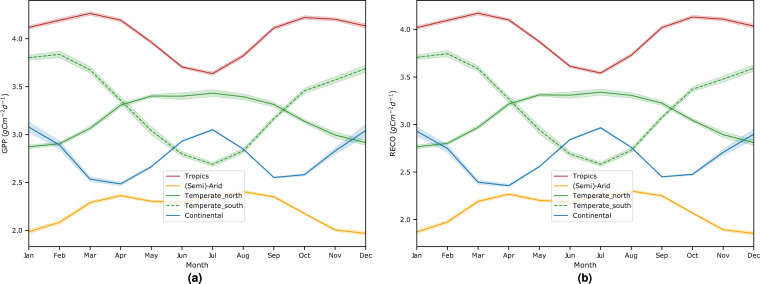
Seasonality of (**a**) GPP and (**b**) *R*_*eco*_ across climate zones for the years 2001–2021.

First, we analyze the seasonality of our upscaled GPP and *R*_*eco*_ across months for the years between 2001 and 2021. As shown in Fig. 9, both fluxes exhibit similar seasonality albeit at different magnitudes. The tropics (including the dry and wet regions) contribute the most to the global GPP and *R*_*eco*_, as expected^39,40^, while the semi-arid regions contribute the least^41^. Carbon fluxes in the temperate (northern hemisphere) and continental regions exhibit unimodal variations that peak in the summer (June, July, and August - JJA), while those in the southern temperate regions peak in December, January, and February (DJF)^42^. On average, the temperate regions have higher carbon fluxes than the continental areas, which tend to be limited by light and temperature, with shorter growing seasons^43^.

**Fig. 10:**
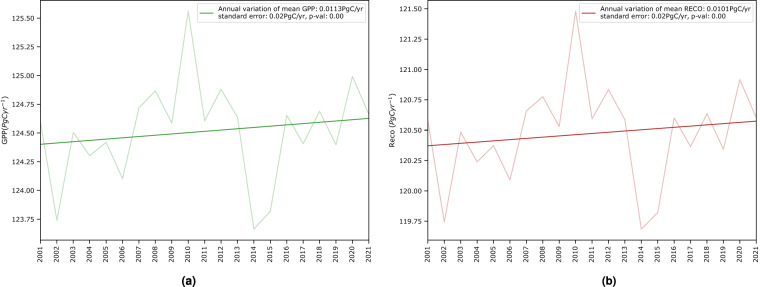
Interannual trends and variations of global (**a**) GPP and (**b**) *R*_*eco*_ for 2001–2021. Overall we observe an increase of 0.0113 *PgCyr*^−1^ (standard error: 0.02 *PgCyr*^−1^) and 0.0101 *PgCyr*^−1^ (standard error: 0.02 *PgCyr*^−1^) for both fluxes, respectively. At a critical value of 0.01, both regression lines are significant with with p-value less than 0.01.

**Fig. 11:**
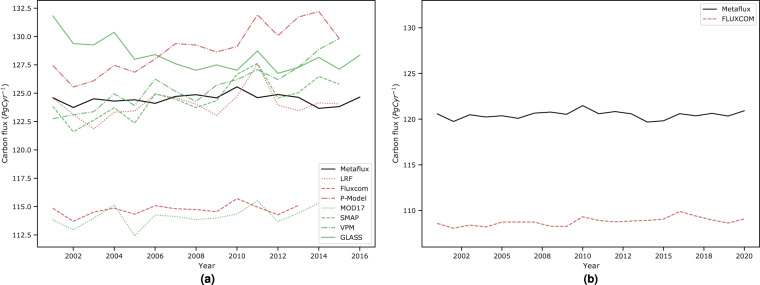
Interannual global average of (**a**) GPP and (**b**) *R*_*eco*_ (*PgCyr*^−1^) in comparison with other global carbon products.

Another interesting analysis is to understand the long-term trends of our global carbon fluxes. As observed in Fig. 10, our meta-trained global carbon product shows an overall increase in GPP by 0.0113 *PgCyr*^−1^ and *R*_*eco*_ by 0.0101 *PgCyr*^−1^. We extend Fig. 10 by making a comparison with other carbon flux products, including those from light response function (LRF)^44^, P-model^45^, MODIS17 (MOD17)^46^, Soil Moisture Active Passive (SMAP)^47^, vegetation photosynthesis model (VPM)^27^, and Global LAnd Surface Satellite (GLASS)^48^ for GPP, as well as Fluxcom^29^ for both GPP and *R*_*eco*_. Overall, they show similar peaks and declines, albeit at varying magnitudes (between 100–140 *PgC yr*^−1^) as shown in Fig. 11.

#### Interannual variability

**Fig. 12:**
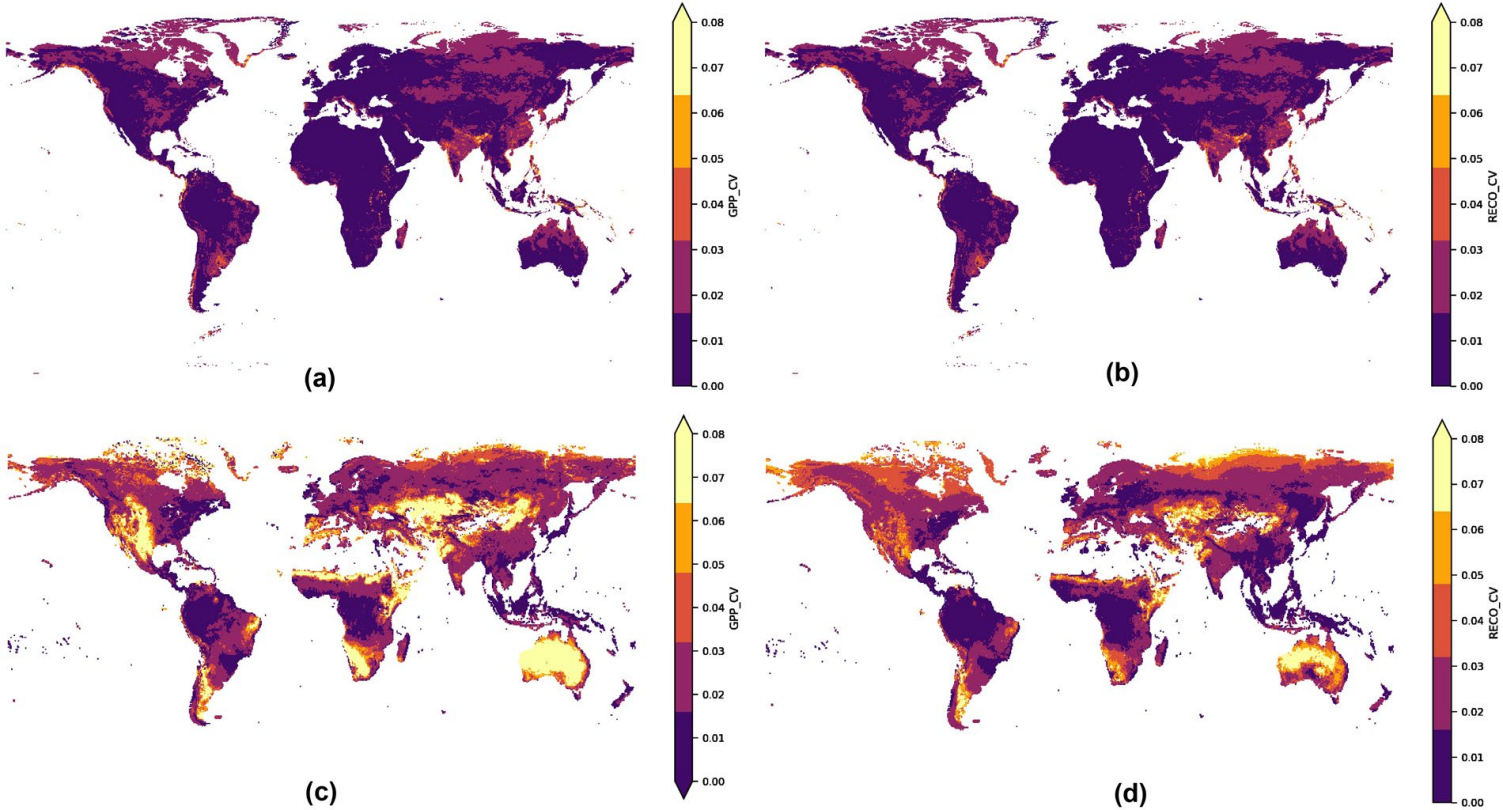
The interannual variability as measured by the coefficient of variation (CV) for (**a**) MetaFlux GPP, (**b**) MetaFlux *R*_*eco*_, (**c**) Fluxcom GPP, and (**d**) Fluxcom *R*_*eco*_.

**Fig. 13:**
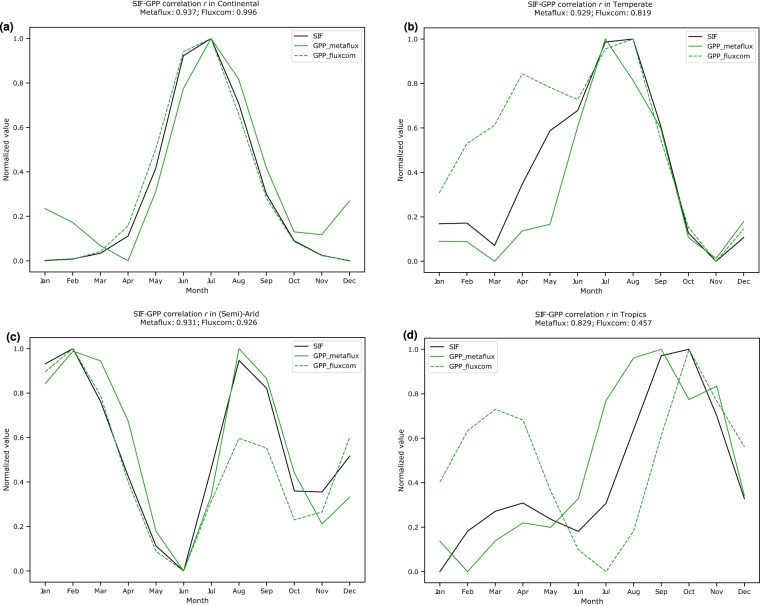
Aggregate Pearson correlation coefficient for the seasonality of *GPP*_*metaflux*_ (versus *GPP*_*fluxcom*_) and CSIF for the year 2012 in the (**a**) continental – 0.937 (versus **0.996**), (**b**) temperate – **0.929** (versus 0.819), (**c**) semi-arid – **0.931** (versus 0.926), and (**d**) tropics – **0.829** (versus 0.457). The y-axis represents the min-max scaled value of either GPP or SIF for that particular climate zone.

**Fig. 14:**
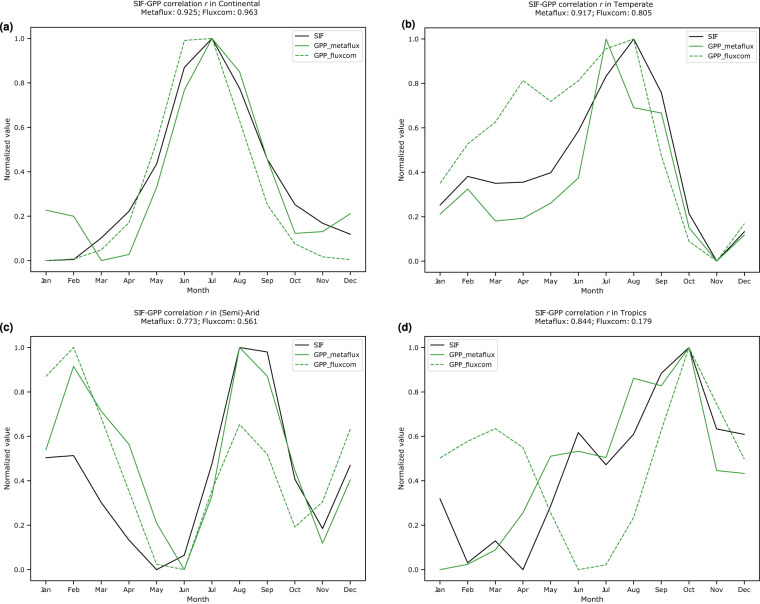
Aggregate Pearson correlation coefficient for the seasonality of *GPP*_*metaflux*_ (versus *GPP*_*fluxcom*_) and TROPOSIF for the year 2019 in the (**a**) continental – 0.925 (versus **0.963**), (**b**) temperate – **0.917** (versus 0.805), (**c**) semi-arid – **0.773** (versus 0.561), and (**d**) tropics – **0.844** (versus 0.179). The y-axis represents the min-max scaled value of either GPP or SIF for that particular climate zone.

We find that the semi-arid regions of Australia, America, and some parts of the northern latitudes have the largest interannual variability of GPP and *R*_*eco*_ (Fig. 12). This is consistent with results from^2^ and^3^ that reported significant contribution of these regions, particularly of the Australian ecosystems, in explaining much of the global carbon interannual variability. As a result, the high turnover rate of carbon pools in these semi-arid environments warrants further research into how the climate and anthropogenic factors can account for this large interannual variability, such as the extent of carbon stock decomposition (e.g. due to wildfire) and accumulation during the dry and wet seasons. In addition, our upscaled product shows high interannual variability in the dry tropical regions of Asia. However, this variation becomes smaller in the tropical forests of Asia, Africa, and America owing to their relatively stable climate. This can be attributed to the region’s sensitivity to rainfall pattern driven by El Niño-Southern Oscillation (ENSO), or soil moisture^49,50^ and rapid land-use changes^51^. In contrast to Fluxcom, our upscaled product does not show as much interannual variability, especially in desert regions (eg. Australia, Central America, South America, and Central Asia), which may be more accurate owing to the extremely low primary productivity there in the first place^52^. Nonetheless, we note that in some parts of the globe, especially along the Sahel and continental Western Europe, the interannual variability of carbon fluxes from MetaFlux is smaller. Physically, this phenomenon has been reported by^53^ and^54^ who observe how variations in terrestrial carbon productivity tend to be stronger in space rather than time. The second plausible reason would be that the ensemble captures much of this variability (i.e. expressed as standard deviation), where each member model learns a different temporal structure that can result in lower than expected mean interannual variability. Lastly, and as highlighted in Figs. 13, 14, meta-learning attempts to learn efficiently from historically underrepresented regions, such as the tropics, which tend to have low interannual variability. This potentially results in a reduction in such variability at higher latitudes, especially along the temperate and continental regions.

#### Comparison with Solar-induced fluorescence (SIF)

In order to evaluate the quality of the seasonal cycle of our product, in particular GPP, we measure its correlation coefficient with several SIF products. MetaFlux GPP demonstrates higher Pearson correlation coefficient with both CSIF and TROPOSIF (Figs. 13, 14, Tables 4, 5) than Fluxcom GPP across the temperate, semi-arid, and tropical regions with values higher than 0.8 to 0.9 even at the very northern latitudes. In particular, the correlation coefficient of our upscaled product with TROPOSIF in the semi-arid, tropics, and temperate regions are 0.856 ± 0.083 (versus 0.726 ± 0.165), 0.546 ± 0.299 (versus 0.343 ± 0.164), and 0.919 ± 0.002 (versus 0.826 ± 0.021), respectively. A similar trend is also observed in the CSIF case where the correlation in the semi-arid, tropics, and temperate regions are 0.925 ± 0.026 (versus 0.914 ± 0.060), 0.772 ± 0.080 (versus 0.608 ± 0.105), and 0.922 ± 0.022 (versus 0.914 ± 0.037), respectively. Across the two SIF products, however, we observe weaker correlation strength in the continental regions. Upon further inspection of Figs. 13, 14, the weaker association could be due to the slight uptick of our GPP estimate during the DJF period, which can be attributed to the lower quality of LAI retrievals because of snow cover.

**Table 4:** Mean Pearson correlation coefficient for the seasonality of GPP (MetaFlux and Fluxcom) and CSIF in the years 2001-2018 across climate zones.

Climate zones	r(*GPP*_*fluxcom*_, SIF)	r(*GPP*_*metaflux*_, SIF)
Semi-arid	0.914 ± 0.060	**0.925 ± 0.026**
Continental	**0.994 ± 0.002**	0.944 ± 0.008
Temperate	0.914 ± 0.037	**0.922 ± 0.022**
Tropics	0.608 ± 0.105	**0.772 ± 0.080**

The numbers in bold represent product with higher GPP-SIF correlation.

**Table 5:** Mean Pearson correlation coefficient for the seasonality of GPP (MetaFlux and Fluxcom) and TROPOSIF in the years 2019–2020 across climate zones. The numbers in bold represent product with higher GPP-SIF correlation.

Climate zones	r(*GPP*_*fluxcom*_, SIF)	r(*GPP*_*metaflux*_, SIF)
Semi-arid	0.726 ± 0.165	**0.856 ± 0.083**
Continental	**0.965 ± 0.002**	0.937 ± 0.012
Temperate	0.826 ± 0.021	**0.919 ± 0.002**
Tropics	0.343 ± 0.164	**0.546 ± 0.299**

**Fig. 15:**
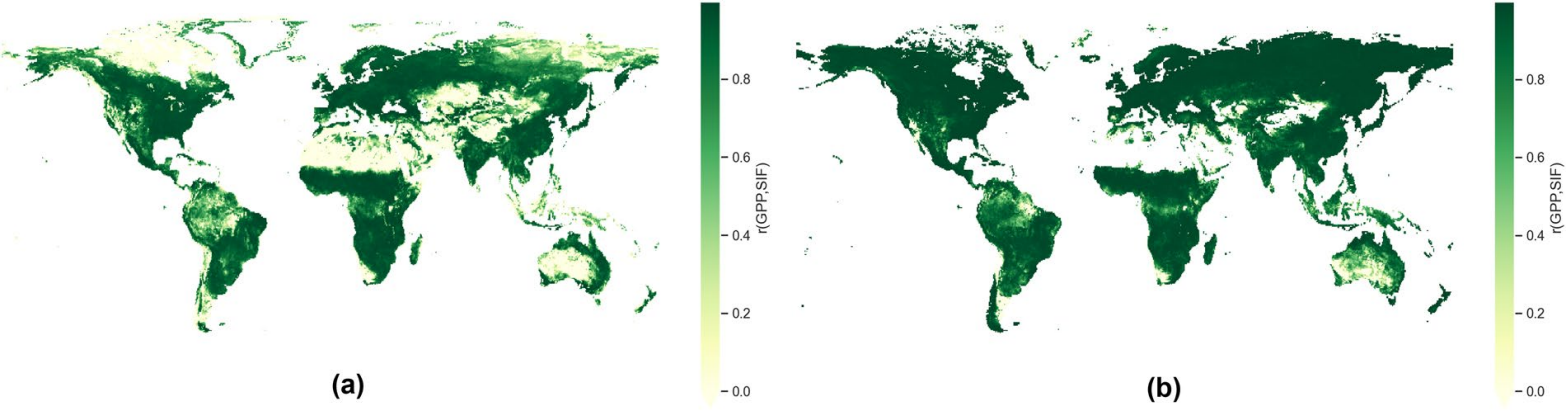
Pixel-level Pearson’s correlation coefficient between the seasonality of SIF and GPP for (**a**) MetaFlux and (**b**) Fluxcom.

Finally, we inspect the pixel-level correlation distribution of MetaFlux and Fluxcom with long-term CSIF product, as illustrated in Fig. 15. In general, MetaFlux has lower correlation with SIF in the tropical rainforest of Indonesia and Amazon as well as the arid regions of Australia, Gobi, Arabian, Syrian, Karakum, Taklamakan, Gobi, and the Great Plains in Northern America. This trend is consistent with earlier reports by^55^, for example, who showed how arid and extremely wet tropical regions (e.g. rainforests) tend to have low GPP-SIF correlation because of weak seasonality that essentially drop correlations to background noise level.

In summary, we have developed a new terrestrial carbon flux product, MetaFlux, using an ensemble of meta-learned deep networks. We have demonstrated how meta-learning can better estimate fluxes in data-sparse, yet critical regions (e.g. semi-arid and the tropics) and are more robust to predicting extreme observations. Our global product is able to outperform other reference product when evaluated against independent measurement, such as SIF or on flux tower networks. We believe that although data sparsity can be a major limiting factor to our complete understanding of many climate processes, leveraging knowledge in other similar domains can be powerful to better understand processes and their response to the environment.

## Usage Notes

The data is permanently stored in Zenodo at 10.5281/zenodo.7761881^12^ and is available at either the monthly or daily temporal scale. Each file contains four variables, GPP, *R*_*eco*_, GPP_std, and *R*_*eco*__std, that are resolved continuously at a 0.25-degree spatial resolution. We purposely mask out the cold regions of Antarctica and the Arctic circle because we assume the lack of GPP and *R*_*eco*_ there. Although we do not mask out the arid regions (e.g. deserts), we would recommend users to do so in order to remove any artifical, though small, estimates. In addition, we do not estimate net ecosystem exchange (NEE) explicitly. One of the primary reasons is because their fluxes (and by extension, their magnitude of variability) are significantly lower than that of GPP and *R*_*eco*_, making estimations from current input variables difficult and because the underlying drivers of GPP and *R*_*eco*_ can differ. Separating the fluxes will ensure better generalization across regimes. Nonetheless, since we use the night-time partitioning algorithm that extrapolates respiration-based NEE estimates (where GPP is assumed to be absent during night-time) to daytime^56^, users are able to get an approximation of NEE by subtracting GPP from *R*_*eco*_ (i.e., *R*_*eco*_ - GPP). However, this approximation is still subject to broader validation, which we leave for future work.

## Data Availability

The meta-learning code is freely available and accessible at https://github.com/juannat7/metaflux. The repository contains notebooks that are customizable to one’s needs beyond the scope of this work. Further questions, feedback, or comments can be directed to the corresponding author.
